# Lens stem cells may reside outside the lens capsule: an hypothesis

**DOI:** 10.1186/1742-4682-4-22

**Published:** 2007-06-08

**Authors:** Susann G Remington, Rita A Meyer

**Affiliations:** 1 Ophthalmology Research, HealthPartners Medical Group and Research Foundation, Regions Hospital, 640 Jackson Street, St. Paul, MN 55101, USA; 2 Department of Biomedical Sciences, Creighton University, Criss I, Room 217, 2500 California Plaza, Omaha, NE 68178, USA

## Abstract

In this paper, we consider the ocular lens in the context of contemporary developments in biological ideas. We attempt to reconcile lens biology with stem cell concepts and a dearth of lens tumors.

Historically, the lens has been viewed as a closed system, in which cells at the periphery of the lens epithelium differentiate into fiber cells. Theoretical considerations led us to question whether the intracapsular lens is indeed self-contained. Since stem cells generate tumors and the lens does not naturally develop tumors, we reasoned that lens stem cells may not be present within the capsule. We hypothesize that lens stem cells reside outside the lens capsule, in the nearby ciliary body. Our ideas challenge the existing lens biology paradigm.

We begin our discussion with lens background information, in order to describe our lens stem cell hypothesis in the context of published data. Then we present the ciliary body as a possible source for lens stem cells, and conclude by comparing the ocular lens with the corneal epithelium.

## Background

### Lens background

**Figure 1:**
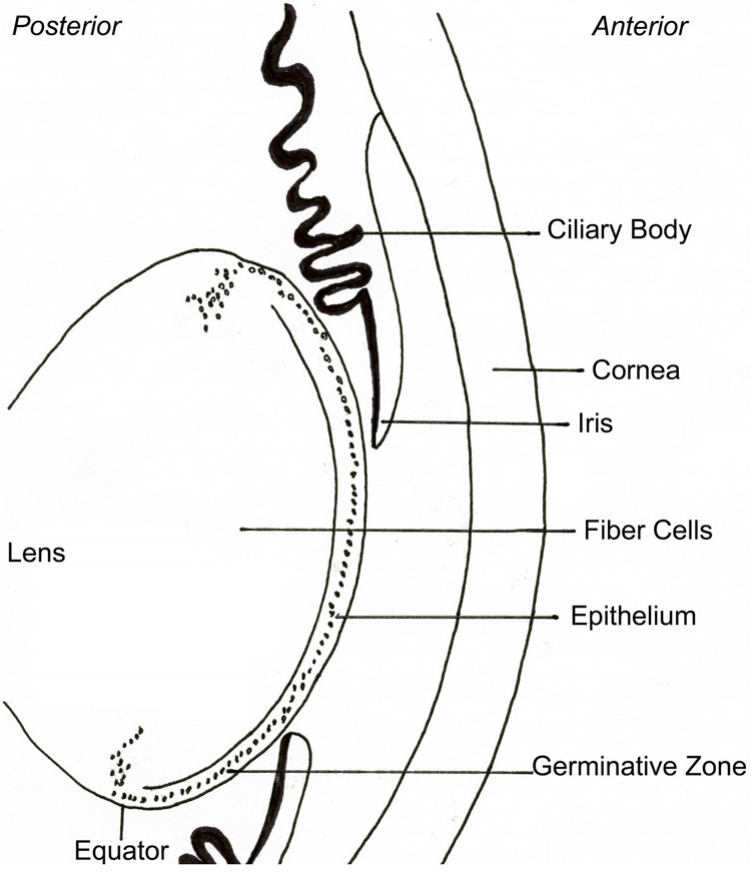
Lens and anterior eye. Cross sectional diagram of the anterior portion of a developing vertebrate eye, based on a 13-day embryonic chicken eye section (photomicrograph of Sandra Ackerley, University of Guelph).

The vertebrate lens is a transparent cellular structure, specialized to focus and transmit light. The lens is composed of two cell types – epithelial cells that form a single cuboidal layer on the anterior surface, and elongated fiber cells that form the posterior bulk of the lens (Figure 1). A capsule of extracellular matrix components encompasses the lens.

The lens grows slowly throughout life, primarily via cell division in the germinative zone. The germinative zone is a narrow cellular region that rings the lens epithelium toward the periphery of the anterior lens surface. Newly formed cells within the germinative zone elongate and migrate along the inner capsular surface toward the lens equator, forming new lens fiber cells as they continue to elongate and migrate posteriorly beyond the equator. These new fiber cells add to the periphery of the existing fiber cell mass, displacing older fiber cells toward the interior of the expanding lens [1-3]. Central fiber cells are retained for life. Historically, the adult lens has been viewed as a closed system, in which all lens precursor cells or stem cells reside within the capsular confines.

### Lens stem cells

We use the following definition of lens stem cells – cells with prolonged self-renewing capacity, that produce one or more differentiated cell types with limited proliferative capabilities [4,5]. In general, stem cells are small, undifferentiated cells that reside in contact with a basement membrane in a protected location known as a stem cell niche. Infrequent stem cell divisions result in one of two cell outcomes. The new cell either remains in its niche as a stem cell, or leaves as a progenitor cell that migrates from the niche to participate in cell differentiation events. Progenitor cells destined for differentiation increase in number through multiple, finite cell divisions as transit amplifying cells [5-7].

A lifetime of cell division in the lens implies the existence of a lens stem cell population. Typically stem cells reside in a protected niche, which for surface or exposed epithelia is a pigment protected and well vascularized location [8,9]. The lens lacks both pigment and a vascular system. An additional point is that tumors often arise from stem cells [10,11], yet the lens does not develop tumors [12,13].

How might these incongruities be reconciled? We hypothesize that the lens is not a closed system. Specifically, lens stem cells may reside outside the lens capsule. If the adult lens does not contain its own stem cell population, we asked where lens stem cells could exist. The pigmented, vascularized ciliary body lies in close proximity to the lens germinative zone, located outside of the lens capsule [14-17]. We propose that the ciliary body could serve as a potential source of stem cells for the lens. We will discuss the ciliary body in more detail below.

## Discussion

### Cell proliferation in the lens

Cells in the lens germinative zone divide throughout life, albeit less frequently with advancing age [14]. Newly divided cells differentiate into fiber cells and add to the periphery of the posterior fiber cell mass. Anterior epithelial cells, if they replicate under normal circumstances, do so infrequently [1-3].

Several observations have supported the idea that lens is a self-contained developmental system. The lens is physically separate from other ocular tissues, and surrounded by a thick capsule of extracellular matrix. The lens is suspended in the eye orbit from the ciliary body by zonular fibrils anchored in the lens capsule. Only two cell types, lens epithelial cells and lens fiber cells, are found within the intact lens. There are no nerves, no blood vessels, and no immune cells within the lens capsule [18,19].

DNA-labeling studies demonstrated that most new lens cells arise in the germinative zone, with a few new cells scattered in the anterior epithelium [14,20-22]. If the lens is a closed system, lens stem cells must reside either in the anterior epithelium or in the more peripheral germinative zone, the only two lens regions with cells that synthesize DNA. As the most rapidly proliferating region and the immediate source of differentiating fiber cells, the germinative zone was often assumed to harbor stem cells for the lens [23]. In support of this argument, the cells in the germinative zone are protected from direct UV radiation by the pigmented iris.

In contrast, cells of the central lens epithelium are exposed to UV radiation that traverses the cornea and aqueous humor. Only a small amount of UVB (the principal DNA damaging wavelengths) reportedly reaches the anterior lens [24], however damage sustained by lens cells could be cumulative [25-27]. A recent long term DNA-labeling study [22] identified the central lens epithelium as the site of the slowest cycling cells in the lens (discussed in more detail below).

Regardless of the actual lens stem cell location, short term labeling studies indicate that the transit amplifying population for the lens resides in the germinative zone. Many transit amplifying cell progeny migrate toward the equator and ultimately differentiate into fiber cells [14,15,21]. Do some transit amplifying cells also migrate centripetally and provide new lens epithelial cells?

### Lens cell lineage

If cell migration occurs within the anterior portions of the lens epithelium, the direction of this migration has not been conclusively determined. There is some circumstantial support (enumerated below) for transit amplifying cells of the germinative zone to supply precursors of new epithelial cells, as well as fiber cells. 1) As organisms age, the volume of the lens increases through new fiber cell addition at the lens equator. The growing lens maintains an epithelial cell monolayer over its expanding anterior surface area. While individual lens epithelial cells increase in average size with advancing age, some epithelial cell division is required to maintain the observed cell coverage [23]. New cells are needed in particular toward the periphery of the anterior epithelial region. Transit amplifying cells of the germinative zone are well positioned to fill this need. 2) Apoptosis of lens epithelial cells has been observed in normal and cataractous lenses [28,29]. Extrapolation of estimated apoptosis rates and cell division rates in the central epithelium suggests that replacement epithelial cells originate toward the lens epithelial periphery and migrate centripetally. 3) Injury of cells in the central lens epithelium resulted in increased DNA synthesis within 24 hours in the lens germinative zone. At later time points (four days), DNA synthesis was also observed in more central epithelial cells surrounding the wound [30]. One possible interpretation of these central epithelium wounding studies is that cells from the germinative zone may routinely migrate centripetally to replace damaged epithelial cells. By analogy, limbal cells are the recognized source of new corneal epithelial cells, and central corneal wounding was demonstrated to stimulate limbal cell proliferation [31-33]. 4) In vitro lens cell migration studies performed in an electric field provided indirect support for centripetal migration of lens epithelial cells in vivo [34]. 5) Several other researchers have proposed centripetal migration of lens epithelial cells based on their own diverse experimental observations [35-38].

If transit amplifying cells in the germinative zone provide replacement cells for the anterior epithelium, then cells of the germinative zone would possess differentiation potential for two different lens cell types – epithelial cells and fiber cells. Individual cells may have the potential to differentiate either as epithelial or fiber cells. Alternatively, two distinct precursor cell populations may reside within the lens germinative zone.

### Lens stem cell hypothesis

While circumstantial evidence implicates the germinative zone as the source of new cells for lens epithelium as well as for fiber cells, results from a recent study seem to contradict these ideas. Long term DNA-labeling experiments demonstrated that central lens epithelial cells retained label longer than cells in the lens germinative zone [22]. By analogy with stem cell studies in other adult tissues, the lens cells that retained label for the longest time periods should include the lens stem cell population. If the lens is a closed system, then this experimental evidence suggests that lens stem cells reside in the central epithelium. However, the central lens epithelium lies in the path of UV radiation, an exposed position for a stem cell population from the standpoint of potential DNA damage.

We propose another possible interpretation for long term labeling of cells in the central lens epithelium. If lens stem cells reside outside the capsule, putative lens stem cells would not have been included in the analyses. The heavily labeled central epithelial cells could simply represent cells that had not divided during the course of the experiment, supporting the view that lens epithelial cells divide very infrequently [14,29,39,40]. (Mature fiber cells, which are maintained for life, lose their cell nuclei and hence are not labeled in long term studies.) Since no heavily labeled cells in the lens germinative zone were observed after 12 weeks, one can infer that slow cycling lens stem cells do not reside in the germinative zone. We hypothesize that lens stem cells reside outside the capsule.

### Ciliary body, a possible source of lens stem cells

If the encapsulated lens does not contain its own stem cell population, we asked where lens stem cells could reside. The ciliary body is a pigmented and vascularized tissue, that lies physically close to the lens germinative zone [14-16,41]. The ciliary body represents the anterior extension of the choroid, and is situated between the choroid and the iris. The epithelium of the ciliary body consists of two cell layers, an inner non-pigmented epithelium, and an outer pigmented epithelium in intimate contact with capillaries [16]. The ciliary epithelial layers represent anterior extensions of the inner non-pigmented neural retina and the outer pigmented retinal epithelium, respectively. (The terms 'inner' and 'outer' are used in reference to the ocular globe interior.) A recognized stem cell population – the retinal stem cells – resides in the ciliary body [42-44].

At early stages of eye development, the presumptive ciliary body abuts the lens capsule overlying the germinative zone [41,45,46]. As the eye matures, the ciliary body elaborates radial processes, each consisting of the double layered epithelium surrounding a central capillary. Extracellular zonular fibrils extend from the posterior ciliary body and the valley walls and floor of the ciliary processes to the equatorial lens capsule, suspending the lens in the eye orbit. The anterior zonular fibrils insert in the lens capsule in a ring near the lens germinative zone [47]. In the primate adult, the inward extensions or 'hills' of the convoluted ciliary body processes lie within one or two millimeters of the lens capsular surface overlying the lens germinative zone [16,48]. During accomodation, the ciliary process 'hills' can contact the lens capsule [48,49].

If the ciliary body harbors lens stem cells, then cells within the ciliary body must satisfy two criteria (discussed in more detail below). 1) Some cells must have the potential to differentiate into lens fiber cells, and 2) ciliary body cell progeny must migrate to the lens as lens progenitor cells. We use the term 'lens progenitor cells' to denote stem cell progeny that will differentiate into lens epithelial or fiber cells.

1) In support of lens fiber cell differentiation potential, ciliary body and other pigmented tissues of the eye have the capacity to develop lentoids in culture [50-54]. Lentoids are groups of cells that express lens fiber cell proteins, such as crystallins, and exhibit lens fiber cell features, such as enlarged transparent cytoplasm. We surmise that the 'retinal' stem cell population could include stem cells with the potential to differentiate into lens.

Another phenomenon – lens regeneration in the newt – also supports the concept of an extracapsular or extralenticular source of lens progenitor cells. Within a few days after loss of the ocular lens in adult urodeles, a new lens begins to emerge from the pigmented iris [55-57]. In both lentoid formation and lens regeneration, the mechanism has been attributed to transdifferentiation of pigmented epithelial cells [56,58]. While we favor ciliary body stem cells as a potential source of lens progenitor cells, transdifferentiation would be a compatible mechanism.

2) The second criterion for the existence of extracapsular lens stem cells involves cell migration. During development, the presumptive ciliary body abuts the lens capsule [41,45,46]. Early migrating lens progenitor cells would have to exit the ciliary body and traverse the immature lens capsule overlying the lens germinative zone. In the adult eye, migration of cells from the ciliary body to the lens would require committed lens progenitor cells to traverse a short acellular distance of aqueous humor between the ciliary body and the lens, as well as traverse the extracellular matrix of the capsule.

Cell migration is an integral part of developmental systems. In the corneal epithelium for example, limbal stem cell progeny migrate centripetally to populate the corneal surface [59-61]. In the case of the lens, extracapsular lens progenitor cells would need to traverse the aqueous humor in the vicinity of the zonular fibrils. If prospective migrating cells require a physical scaffold for migration, support could be provided by the zonular fibrils, which reach the lens from the valleys of the convoluted ciliary processes [47,62,63]. (For example, cell migration occurs along extracellular matrix fibrils during cardiac development [64]).

The lens capsule itself may provide a formidable cell migration barrier along much of its surface area, however, entry to the lens capsular interior would need to occur only in a limited area near the germinative zone. The lens capsule is not uniform. It differs in thickness and composition between the anterior and posterior surfaces [65-68]. Additional compositional differences near the germinative zone can be inferred from lectin labeling studies [66]. Zonular fibrils interdigitate into the lens capsule structure in the vicinity of the germinative zone [62,69,70]. Zonular fibril tracks might provide lens capsule entry points, as well as a cell migration substrate. We speculate that extralenticular cells could have access to the lens capsule interior via zonular fibril tracks. We are not aware of experimental data to support cell migration into a lens possessing an intact capsule.

### Posterior capsule opacification

If the continuity of the lens capsule is breached, however, extralenticular cell migration into the area delimited by the lens capsule likely occurs. Cataract extraction disrupts the lens capsule. Subsequent cell growth and migration on the remaining capsule lead to complications in 25% of adult patients (and nearly 100% of pediatric patients) that again compromise vision [71-73]. These complications, known as after-cataract or posterior capsule opacification, are believed to primarily involve proliferation and migration of lens epithelial cells left behind during cataract surgery [74-77]. There is also evidence that cells originating in non-lens ocular tissues participate in cell aggregates within the remaining capsule [78-80].

In posterior capsule opacification, the majority of aberrant cell growth is attributed to lens cells originating within the capsule. However, if our hypothesis is correct that lens stem cells normally reside outside the lens capsule, then much of this aberrant growth may actually arise from lens progenitor cells that migrate to the capsule after the cataract surgery.

### Analogies to corneal epithelium

If our lens literature summary seems contrived to explain an improbable lens stem cell hypothesis, consider the corneal epithelium. Like the lens, the corneal epithelium is a transparent, avascular ocular tissue, specialized to focus and transmit light [81]. One major difference between cornea and lens is that the cornea also provides a protective surface for the eye. In its protective role at the environment interface, the corneal epithelium has well developed tissue replacement capabilities to repair normal wear and minor injuries [82,83]. In contrast, lens cell division occurs on a more limited scale.

Corneal epithelial stem cells reside in the limbus, a pigmented and vascularized tissue that inhabits the peripheral boundary of the cornea at its junction with the conjunctiva [31,33,84-86]. The intact limbus forms a barrier to the migration of cells from the adjacent conjunctival epithelium [87,88]. Committed corneal epithelial cells originate in the limbus and migrate centripetally along the basal lamina to populate the basal layer of the corneal epithelium [61]. In the cornea, basal cells represent transit amplifying cells, which continue to divide providing renewed layers of differentiated corneal epithelium [6,89]. Basal corneal epithelial cells rarely, if ever, beget tumors, despite their ability to replicate their DNA and divide. Most tumors observed in the cornea originate in the limbus and grow to impinge on adjacent corneal tissue [11,90,91].

There are many similarities between the established biology of the corneal epithelium, and our hypothesized source of lens stem cells. Analogous to the cornea, we propose that lens stem cells reside in a protected location – the pigmented, vascularized ciliary body. Non-lens cells do not indiscriminately migrate through the lens capsule. However, committed lens progenitor cells would need to migrate to the lens inner capsular surface (basal lamina) to populate the germinative zone. Transit amplifying cells in the lens germinative zone would subsequently differentiate and migrate as new fiber cells along the inner surface of the equatorial capsule. (Other transit amplifying cells could follow an alternative differentiation pathway and migrate centripetally as new lens epithelial cells along the inner surface of the anterior capsule.) Analogous to committed cells in the basal layer of the corneal epithelium, cells in the lens germinative zone continue to replicate their DNA, yet maintain their commitment to lens cell differentiation. Lens cells do not naturally develop tumors.

## Conclusion

In light of concepts that have evolved in stem cell literature in recent years, we re-examine the ocular lens in the context of features common to other biological tissues. Since the lens grows throughout life and does not naturally develop tumors, we ask whether lens stem cells could reside in a more typical stem cell niche, one that is pigmented and vascularized. We hypothesize that lens stem cells reside outside the lens capsule in nearby pigmented ocular tissue, the ciliary body. Here, we present our review of the lens literature from this novel perspective.

We conclude that a postulated extracapsular source of ocular lens stem cells is consistent with a large body of literature. Future experiments on lens development, stem cell biology, cell migration, and ocular oncology may shed light on the robustness of these concepts. In the meantime, we hope that our provocative ideas will stimulate discussion in the fields of lens and ocular biology, and encourage the consideration of experimental results from multiple perspectives.

## Competing interests

The author(s) declare that they have no competing interests.

## Authors' contributions

SGR conceived the hypothesis, researched the literature, and drafted the manuscript. RAM participated in literature research and interpretation, refined the ideas, and helped prepare the manuscript. Both authors read and approved the final manuscript.
